# The influence of spatial and temporal resolutions on the analysis of cell-cell interaction: a systematic study for time-lapse microscopy applications

**DOI:** 10.1038/s41598-019-42475-5

**Published:** 2019-05-01

**Authors:** M. C. Comes, P. Casti, A. Mencattini, D. Di Giuseppe, F. Mermet-Meillon, A. De Ninno, M. C. Parrini, L. Businaro, C. Di Natale, E. Martinelli

**Affiliations:** 1 Department of Electronic Engineering, University of Rome Tor Vergata, Rome, Italy; 2 Institute Curie, Centre de Recherche, Paris Sciences et Lettres Research University, 75005 Paris, France; 3 Department of Civil Engineering and Computer Science, University of Rome Tor Vergata, 00133 Rome, Italy; 4 Institute for Photonics and Nanotechnology, Italian National Research Council, 00156 Rome, Italy

**Keywords:** Biomedical engineering, Electrical and electronic engineering

## Abstract

Cell-cell interactions are an observable manifestation of underlying complex biological processes occurring in response to diversified biochemical stimuli. Recent experiments with microfluidic devices and live cell imaging show that it is possible to characterize cell kinematics via computerized algorithms and unravel the effects of targeted therapies. We study the influence of spatial and temporal resolutions of time-lapse videos on motility and interaction descriptors with computational models that mimic the interaction dynamics among cells. We show that the experimental set-up of time-lapse microscopy has a direct impact on the cell tracking algorithm and on the derived numerical descriptors. We also show that, when comparing kinematic descriptors in two diverse experimental conditions, too low resolutions may alter the descriptors’ discriminative power, and so the statistical significance of the difference between the two compared distributions. The conclusions derived from the computational models were experimentally confirmed by a series of video-microscopy acquisitions of co-cultures of unlabelled human cancer and immune cells embedded in 3D collagen gels within microfluidic devices. We argue that the experimental protocol of acquisition should be adapted to the specific kind of analysis involved and to the chosen descriptors in order to derive reliable conclusions and avoid biasing the interpretation of results.

## Introduction

Direct observation of cell behaviour using light microscopy is certainly one of the least invasive techniques throughout biology to extract meaningful information on living organisms, connecting multiple scales of complexity. A series of technological advances have revolutionized our ability to characterize complex biological events, previously unexplored, including individual and collective cell motion^1,2^, and cell-cell interactions^3–6^. Firstly, microfluidic cell culture chips, with the more recent application in organ-on-chips devices, have enabled recapitulation of physiological multicellular microenvironments at both physical and biochemical levels and have emerged as front-line *in vitro* testing tools for preclinical evaluation of therapeutic agents^7,8^. Secondly, live cell imaging at increased spatial-temporal resolution have allowed monitoring of complex cell behaviours by producing vast amounts of high-content imaging data^9,10^. Lastly, computerized algorithms for cell tracking and analysis have provided the means to automatically detect cell trajectories and quantify relevant motility descriptors^11^.

Within this emerging pipeline for high-throughput experimentation, image-based systems approaches may provide great contributions to dissect cellular interactions in highly complex heterogeneous tissues such as the tumor microenvironment, which have been found to modulate metastasis, angiogenesis and the regulation of immune response. Extraction of quantitative meaningful data from intercellular communication networks in such ecosystems^12^ may be exploited to evaluate the efficacy of anti-tumor immune strategies, including targeted therapies and immunotherapies.

Recently Moore and colleagues^13^ investigated the interactions between tumor biopsy fragments and flowing tumor-infiltrating lymphocytes (TILs) in a dynamic microenvironment. Difference in temporal levels of TILs and tumor death were automatically quantified and correlated to the cell response to immunotherapy. Cancer-stromal interaction was studied by Chen *et al*.^14^ by means of a dedicated microfluidic prototype. By isolating paired cells in a chamber, the authors demonstrated a relationship between the proliferation rate of myoblasts and the pairing ratio of cancer cells. Parlato *et al*.^5^ analysed dendritic cell-cancer cell interactions within a three-dimensional tumour microenvironment on chip. They showed that immunotherapy with interferon-alpha in combination with romidepsin increases the phagocytosis of tumour cells by dendritic cells, resulting in high levels of cell apoptosis. Vacchelli *et al*.^15^ identified a loss-of-function mutation in the allele of the gene coding for formyl peptide receptor 1 (FPR1) that was associated with poor survival in breast and colorectal cancer patients receiving adjuvant chemotherapy. Using a simple microfluidic platform described in Businaro *et al*.^4^, they imaged over time the different migration patterns of immune cells collected from human healthy volunteers carrying all the FPR1 genotypes towards the cancer cells in 2D through microchannels. Experiments confirmed that FPR1 and its ligand, annexin-1 (ANXA1), promoted stable interactions between dying cancer cells treated with anthracyclines and leukocytes, while FPR1 deficiency leads to the immune failure in approaching apoptotic cells leading to defective anticancer immune response and less efficient chemotherapy.

The promising results obtained through the investigation of the dynamic response of cells to external stimuli or chemotactic signalling, have posed the attention to the applicability of numerical descriptors to characterize the cell-cell interactions observed at the microscopic level^6^. Several aspects related to the acquisition and data interpretation protocol contribute to the significance of the derived descriptors in confirming or not the hypotheses on a given experiment. Among them, the set-up of the imaging acquisition device plays a crucial role in determining the performance of the tracking algorithm and, consequently, the efficacy of the kinematic descriptors. Increasing the spatial resolution is desirable to resolve finer details and facilitate automatic cell detection. In addition, higher frame rates allow a more accurate reconstruction of the cell trajectories and of the interaction dynamics from time-lapse videos. However, a trade-off between video quality and high resolution is needed to avoid cell damage due to photobleaching and phototoxicity^16^, as well as to reduce the overall data volume. Moreover, it is important also to remark that an eventual restriction of the field of view to improve an initial low spatial resolution would clearly reduce the number observed cells and then negatively affecting statistical validity of the results. Beltman *et al*.^17,18^ were pioneers in tackling the issue about imaging space and time constraints by estimating interaction time among immune cells during migration. They exhaustively highlighted the main limitations of measuring what they called “contact time” among interacting cells due to frequent artifacts occurring during video acquisition. Mainly, for an unbiased estimation, cell interaction phenomena have to be entirely visualized during the video to avoid underestimation of the contact time. In line with this, we will provide experimental results in support of an accuracy worsening due to a lower frame rate.

Only recently, the pivotal role of the temporal resolution on parameter inference for biological mechanistic models describing biological transport processes, has been investigated by Harrison *et al*.^19^. A Bayesian inference was carried out to estimate posterior distributions of the motility model parameters at increasing time discretization (i.e. at decreasing temporal resolution). From the study, it emerged that spurious characterization of motile behaviour may be avoided thanks to a more rational experimental setting. Anyway, these suggestions regarded the impact of only temporal resolution on a particular motility dynamic referring to specific parameters of the relative mathematical model.

With the aim to present a more general approach to the problem of extracting reliable quantitative descriptors from the motility and cell-cell interaction phenomena, we investigated the effect of both temporal and spatial resolutions on cell tracking software (see Supplementary Text S1) and related kinematics features. Although it is intuitive that the choice of the temporal-spatial resolutions in live cell imaging acquisition is associated with potential artefacts on extracted descriptors, by our knowledge, no one had drawn up an experimental protocol addressed to practitioners providing scientific details to what degree their experimental results may be more or less altered at varying combination of temporal-spatial resolutions.

**Figure 1:**
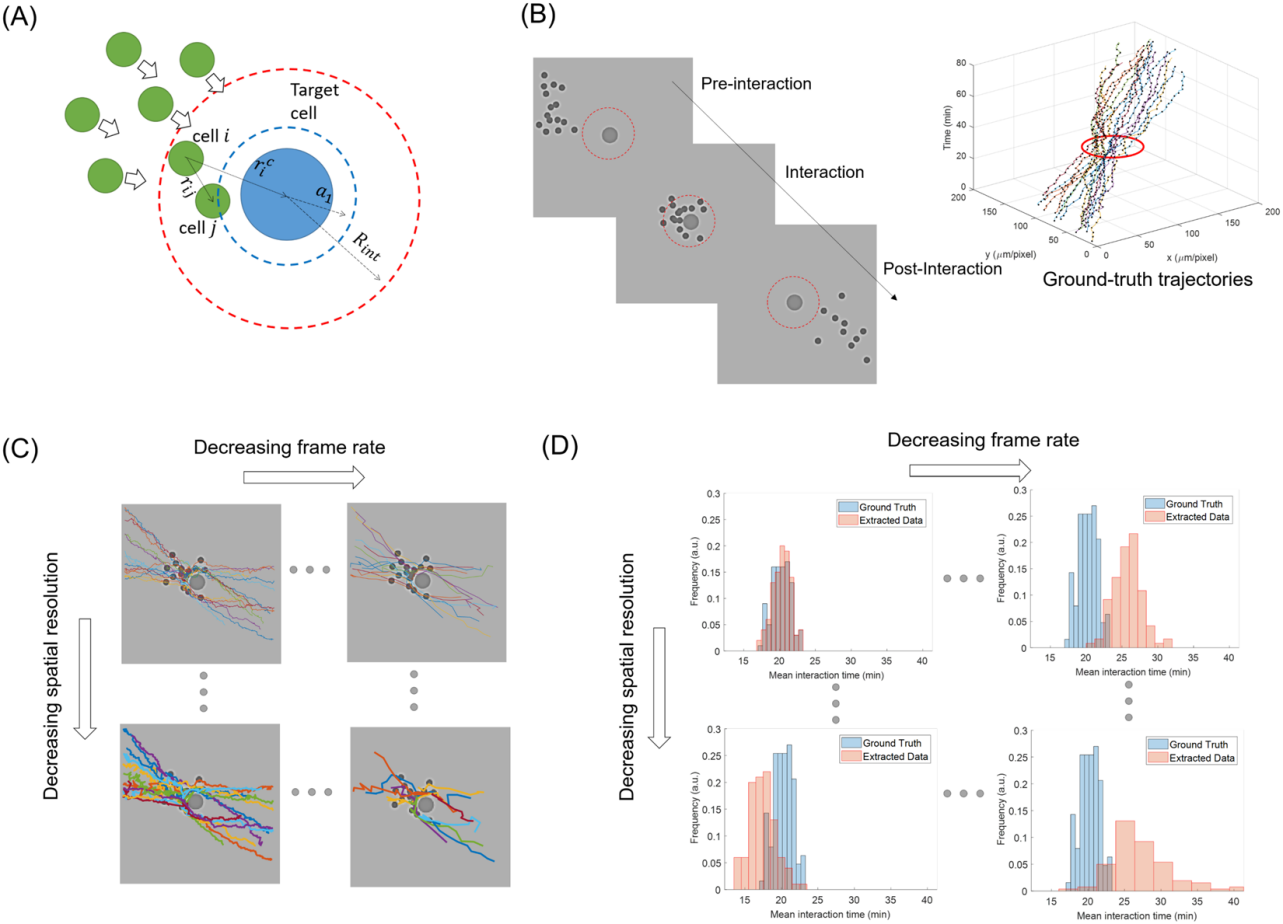
Steps of the analysis platform for the study of the effects of spatial and temporal resolutions on kinematics descriptors. (**A**) Definition of the stochastic particle interaction model. (**B**) Generation of artificial videos. (**C**) Automatic cell tracking at decreasing spatial and temporal resolutions. (**D**) Feature analysis at decreasing spatial and temporal resolutions. Dotted red circles in (**A**) and (**B**) indicate the interaction radius. The steps in (**C**) and (**D**) have been also applied to real experiments based on organ-on-chip devices.

To the aim, we analysed simulated scenarios for a diversified set of experiments, inspired by the work of Vacchelli and colleagues^15^. We first derived a stochastic interacting particle model for immune cells migrating towards and interacting with a target cell (see Fig. 1A). The directed motion of the cells was modelled as a random walk with drift, constant in modulus |***μ***|^3^, so that the probabilistic motion step is biased towards the target as a spontaneous intrinsic process^20^. The physical interactions among cells were modelled through repulsive-attractive potentials^4^. In particular, for the immune-cancer interaction, we imposed an attractive potential in proximity of the target cell for a given effectiveness time, *T*_*eff*_. For different experimental conditions, including a control case scenario and different comparative case studies, we generated an atlas of artificial videos (by varying |***μ***| and *T*_*eff*_), that we downsampled at varying spatial and temporal resolutions (see Fig. 1B). After applying a cell tracking software, *Cell Hunter*^5^ (see Fig. 1C), a set of commonly used kinematic and interaction descriptors was extracted from the detected trajectories (see Fig. 1D): migration speed^5^, persistence^5^, angular speed^21^, Shannon entropy^22^ of angular speed, mean turning angle^3^, ensemble-averaged mean square displacement (MSD)^23^, and mean interaction time^5^. The mathematical expressions of the descriptors are summarized in Table 1. The theoretical trajectories obtained at the maximum spatial and temporal resolutions and the consequent extracted features were used as ground truth for performance evaluation (see Fig. 1B and D respectively).

**Table 1:** List of kinematic and interaction descriptors. The index *i* denotes the *i*^*th*^ track, ${r}_{i}^{l}$ indicates the position of the *i*^*th*^ track at time *l*, *d* is the Euclidean distance, and *L*_*i*_ stands for the length of the *i*^*th*^ track. For the computation of the ensemble-averaged MSD, *l* = 1, …, max(*L*_*i*_).

Kinematics descriptors	
Migration Speed^5^	${v}_{i}=d({r}_{i}^{1},{r}_{i}^{{L}_{i}})/{L}_{i}-1$
Persistence^5^	${p}_{i}=d({r}_{i}^{1},{r}_{i}^{{L}_{i}})/\sum _{l=1}^{{L}_{i}-1}d({r}_{i}^{l},{r}_{i}^{l+1})$
Angular Speed^18^	${w}_{i}^{l}=(\frac{d({r}_{i}^{l},{r}_{i}^{l+1})\,}{\Delta {t}_{i}})\frac{1}{{({r}_{i}^{c})}^{l}}$
Shannon Entropy^19^ of Angular Speed	$E({w}_{i})=-{\sum }_{l=1}^{{L}_{i}}{w}_{i}^{{l}^{2}}\,\mathrm{log}({w}_{i}^{{l}^{2}})$
Mean turning angle^3^	$\theta ={\rm{acos}}(\langle \overline{\cos ({\theta }_{i}^{l+1}-{\theta }_{i}^{l})}\rangle )$
Ensemble-Averaged MSD^20^	$MS{D}_{l}=\langle d{({r}_{i}^{l},{r}_{i}^{l+1})}^{2}\rangle$
**Interaction descriptors**	
Interaction Time^5^	*T*_*int*_: for each target cell, the number of frames in which each immune cell remains within the interaction radius, *R*_*int*_.

The conclusions obtained by the analysis of the artificially generated videos were fully validated by the analysis of videos of real cell-biology experiments, in which unlabelled human cancer cells (the BT474 cell line representative of the HER2+ breast cancer subtype) and immune cells (PBMCs, peripheral blood mononuclear cells, from healthy donors) were co-cultured in 3D collagen gels within microfluidic devices. We show that when the extracted descriptors are used for comparing cell motility in diverse experimental conditions (i.e., not treated versus treated target cells), spatial and temporal resolutions have a direct impact on the overlap between the two compared distributions and on the statistical significance of the difference^24^. With respect to the interaction between immune cells and target cells, we demonstrate that the entropy of the interaction descriptor, at decreasing resolutions, is entwined with the entropy values of pre and post-interaction phases, meaning that the information on the interaction can be completely lost at low resolutions. Accordingly, the influence of experimental resolutions is crucial to investigate the interaction phenomenon and to extract not misleading motility and interaction descriptors.

By providing an extensive characterization of the experimental platform for the analysis of cell-cell interactions in organ-on-chip devices, we show how resolutions can alter their individual discriminative capacity, thus biasing the interpretation of results and the efficacy of the overall experimental platform.

## Results and Discussion

We used innovative computational models and experiments on organ-on-chip devices to study how spatial and temporal resolutions of time-lapse videos influence the quantitative multi-parametric image analysis. The dynamic of intercellular interactions in highly complex biological ecosystems such as tumor microenvironments and the assessment of cell response to drug treatments were inquired. We demonstrated inadequate microscopy set-up procedures may become detrimental, notably, on derived conclusions between experimental conditions in comparison. Actually, biologically relevant differences may be obscured, e.g. the influence of drug administration on immune-cancer interaction time, which is a crucial parameter to estimate the efficacy or not of the therapy. Our results may represent experimental advices to the practitioners in the choice of suitable resolutions in analysing their own experiments. Indeed, they can be generalized to cover a wide spectrum of experiments.

### Analysis of the simulated scenario

**Figure 2:**
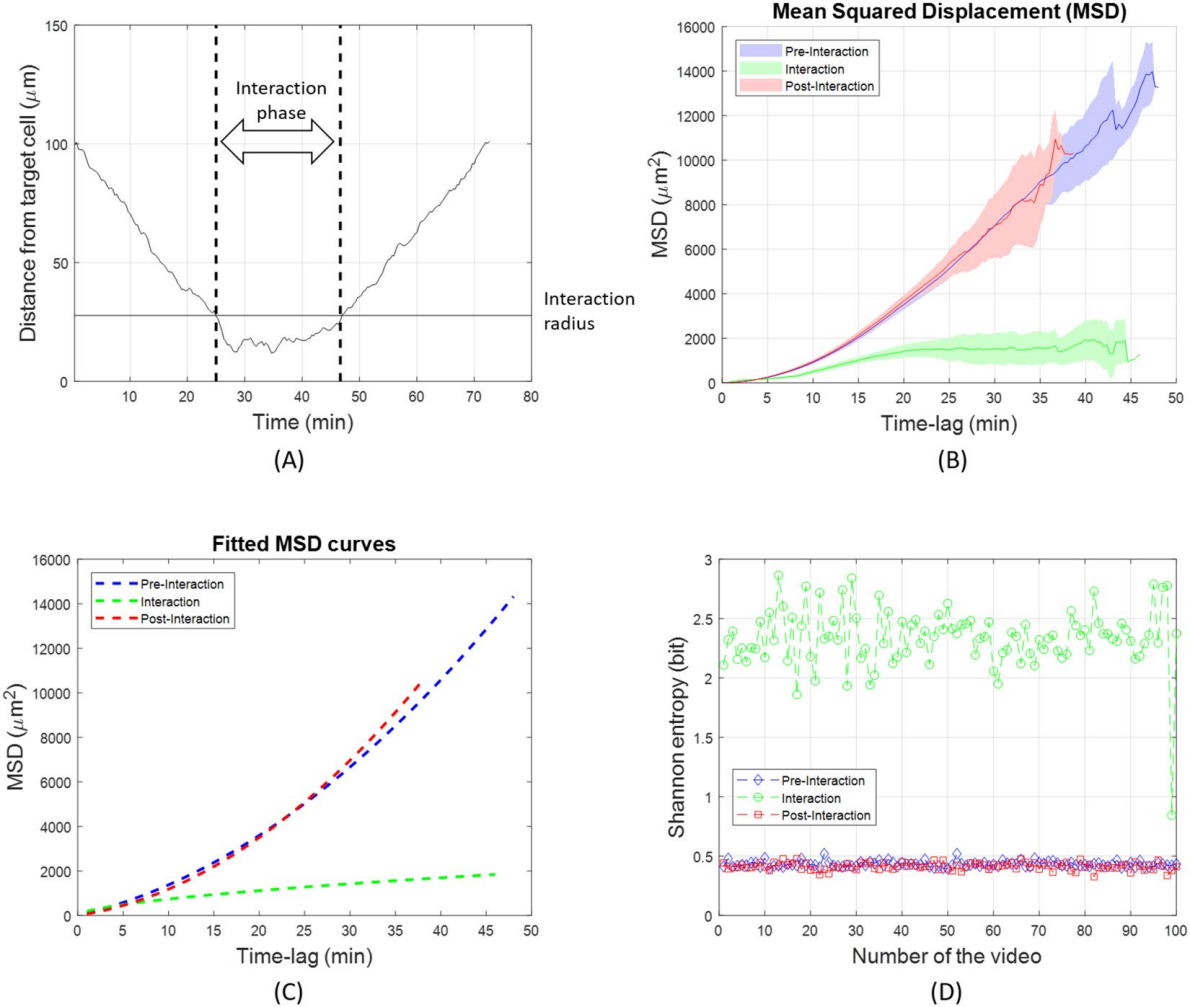
Analysis of the three motion phases. (**A**) Distance of a single cell from the target cell. Dotted lines indicate the extremes of the interaction phase. Based on the interaction radius, three different motion phases are defined: pre-interaction, interaction and post-interaction phase. (**B**) Ensemble-averaged Mean Square Displacement (MSD) curves for the three motion phases as defined in (**A**); mean values and the within standard deviation regions are computed over the 100 simulations. (**C**) Fitted curves of the MSD curves in (**B**) during the three motion phases: pre-interaction (directed Brownian motion), interaction (sub-diffusive motion), post-interaction (directed Brownian motion). (**D**) Shannon entropy of the angular speed averaged over all tracks of the immune cells in each of the 100 videos.

**Figure 3:**
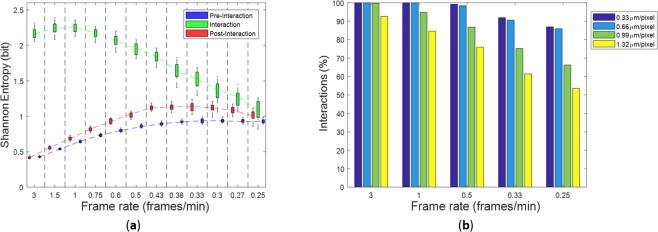
Loss of interaction information at decreasing temporal resolutions when the maximum spatial resolution is fixed. (**a**) Shannon Entropy boxplots for the three phases of motion: pre- interaction (blu), interaction (green), post-interaction (red). (**b**) Percentage of detected interactions between immune cells and the target cells averaged over all simulations.

The study of the interaction between different cell populations is crucial in our study and it is mimicked by the proposed stochastic particle model (see equations (1, 2), Methods). Therefore, we investigated how the interaction phenomenon modified the kinematic behaviour of immune cells. By computing the time-varying distance of each immune cell from the target cell in relation to the interaction radius (see Methods), we were able to distinguish three main motion phases (Fig. 2A): 1) a pre-interaction phase characterized by a directed motion of immune cells towards the target cell; 2) an interaction phase where immune cells were in proximity of the target cell, within the interaction radius; and 3) a post-interaction phase in which immune cells move away from the interaction radius. During interaction with the target, the presence of the target cell forced the immune cells to move in an abnormal diffusion motion (sub-diffusion)^2^, quite different from the random directed motion occurring during the pre- and post-interaction phases. The diffusion anomaly may be observed with the calculus of the ensemble-averaged MSD (Table 1), chosen for our computation because, as a result of a “weak ergodicity breaking”, the time-averaged MSD of a single particle may not convey the desired information^23^. By approximating *MSD*(*t*)~*t*^*α*^, *α* < 1 means anomalous subdiffusion, *α* = 1 normal Brownian diffusion, and *α* = 2 directed motion (random walk with drift)^23,25^. In Fig. 2B the MSD curves computed during the three mentioned motion phases and averaged over all videos are shown. The MSD curve computed during the interaction phase stand out from the other two curves, computed during the pre- and post-interaction phases, whose shapes were very similar to each other. By fitting the average curves shown in Fig. 2B, we obtained the following results:

$$\begin{array}{cc}MS{D}_{pre-int}(t) \sim {t}^{2} & {R}^{2}:0.9930\\ MS{D}_{post-int}(t) \sim {t}^{2}\, & {R}^{2}:0.9951\\ MS{D}_{int}(t) \sim {t}^{0.6} & {R}^{2}:0.8093,\end{array}$$

where *R*^2^ expresses the goodness of fit (Fig. 2C). The change in motion type, i.e., directed-subdiffusion-directed, also influences the Shannon entropy of the angular speed (Table 1), which assumes fairly low and similar values during the two extremal phases, while it increases in the middle phase, in which the angular speed is mostly unpredictable^22^ (Fig. 2D). As spatial and temporal resolutions decrease, the representation provided by the Shannon entropy becomes misleading. At decreasing resolutions, in fact, the entropy of the angular speed during interaction is entwined with the entropy values of pre- and post-interaction phases, as shown in Fig. 3a, that is, the information on the interaction was lost at low resolutions. To confirm this, we observe in Fig. 3b that the percentage of detected interactive cells drastically reduces at lower spatial and temporal resolutions. Accordingly, the influence of experimental resolutions is crucial to investigate the interaction phenomenon and to extract meaningful kinematic and interaction descriptors. In fact, the experimental set-up of time-lapse microscopy, in terms of resolutions, affected the reliability of the cell tracking software (see Supplementary Text S1) and the relative cell-track feature extraction.

Mean and standard deviation maps of the relative error over 100 simulations, expressed in percentage, are represented in Supplementary Fig. S2 for three of the features in Table 1 (ensemble-averaged MSD, interaction time, migration speed) at varying spatial and temporal resolutions. The extracted features deviated from the respective ground-truth values at decreasing resolutions, with average (and standard deviation) values of the relative error up to 50.3%(10.9%), 34.2%(19%), and 40%(6.4%), respectively for ensemble-averaged MSD, interaction time, migration speed. Therefore, working at low resolutions leads to the possibility of unknowingly extracting unreliable descriptors for the experimental scenario.

### Comparison between different experimental conditions in simulated videos at varying spatio-temporal resolutions

**Figure 4:**
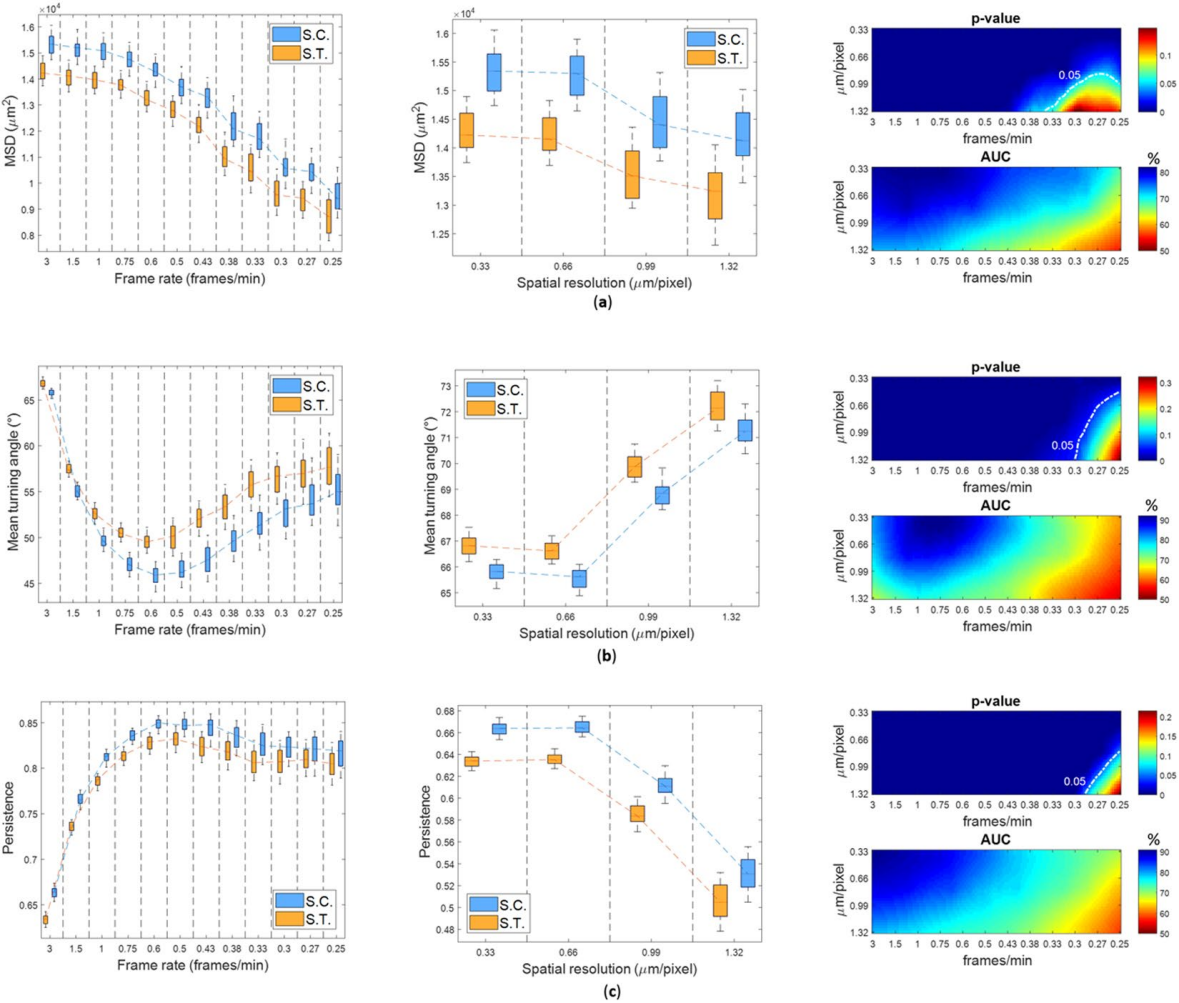
Comparison of diverse experimental conditions for three kinematic descriptors. (**a**) Ensemble-averaged MSD. (**b**) Turning angle. (**c**) Persistence. In the legends S. C. denotes the simulated control (|***μ***| = 3.0 *μm*/*min*, *T*_*eff*_ = 8.3 min), S.T. denotes the simulated treatment:|***μ***| = 3.0 *μm*/*min*, *T*_*eff*_ = 13.3 min. Left panels represent the feature values (mean and standard deviation values) at the maximum spatial resolution (0.33 *μm*/*pixel*) and decreasing temporal resolution. Central panels represent the feature values at the maximum temporal resolution (3 frames/min) and decreasing spatial resolutions. Every region delimited by vertical dotted lines stands for a unique temporal (left panel) or spatial (central panel) resolution. Right panels show the maps of p-values for the K-S test and the area under the receiver characteristic (AUC) curve at varying resolutions (see methods). The white dotted line in the p-value maps indicates the contour line at p = 0.05.

The analysis is now extended to the exploitation of the information content carried out by the cell kinematics for diversified biological experiments. After generating videos of cancer-immune interaction at different experimental conditions by changing some of the kinematic parameters, i.e., effectiveness time and/or drift modulus (see *Numerical Simulations*), we analyzed the combined effect of resolutions on the extracted features. Our goal was to understand the role of time-lapse microscopy setting in determining the ability of the derived kinematic features in discriminating different biological conditions. In particular, as a topic of growing interest in this field^5,15^, we compared scenarios that emulates the effects of targeted therapies on immune cells and on cancer-immune cells interaction. For the scope, we set for a control case, without treatment, (Simulated control in Fig. 4) |***μ***| = 3.0 *μm*/min and *T*_*eff*_ = 8.3 min (see Supplementary Video S1) and for a comparative study case, with treatment, (Simulated treatment in Fig. 4) |***μ***| = 3.0 *μm*/min and *T*_*eff*_ = 13.3 *min* (see Supplementary Video S2). Both scenarios were studied by analysing the extracted kinematic and interaction descriptors (Fig. 4 and Supplementary Fig. S3). The boxplots in the left and central panels of Fig. 4 show the distributions of the features for the simulated control and the simulated treatment obtained for the set of generated videos at decreasing temporal (with maximal spatial resolution) and decreasing spatial resolutions (with maximal temporal resolution), respectively. In addition to a systematic effect of resolutions on the values of the descriptors for each scenario, the degradation of resolutions increases the proximity of the two distributions, thus reducing the difference observed between the two experimental scenarios.

Maps at varying resolutions of the p-values and AUC values obtained for ensemble-averaged MSD, turning angle, and persistence, are shown in the right panels of Fig. 4. The indicators show reduced discriminability and higher p-values at combinations of decreasing resolutions. P-values higher than 0.05 are observed for lower frame rates and spatial resolutions. For the case of persistence, by combining spatial resolutions of [0.99, 1.32] μm/pixel with temporal resolutions of [0.33, 0.30] frames/min, p-values of the order of 10^−3^–10^−4^ indicate a statistical significance of the difference between the simulated control and the simulated treatment, whilst the AUC values indicate a slight discriminatory ability of the descriptor (AUC = [0.60–0.63]). Therefore, the two indicators are in accordance, but they do not lead at identical results. P-value is the measure of statistical evidence par excellence, but it has limits because it is calculated in relation to the null hypothesis only. By involving more extreme data closely related to the experiments under consideration^26^ (in our case the simulated control,the simulated treatment), they could show differences also when discrimination between the two situations is not as evident. The same discussion is valid for the other computed parameters in Table 1 (see Supplementary Fig. S3).

**Figure 5:**
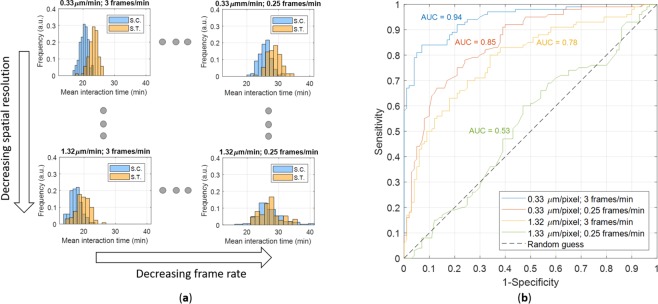
Combined effects of tracking and resolutions on the mean interaction time. (**a**) Histograms for two experimental scenarios at four different combinations of spatial and temporal resolutions. Simulated control (S.C.): |***μ***| = 3.0 *μm*/*min*, *T*_*eff*_ = 8.3 min (in blue). Simulated treatment (S.T.): |***μ***| = 3.0 *μm*/*min*, *T*_*eff*_ = 13.3 min (in red). (**b**) ROC curves illustrating the discriminatory ability of the mean interaction time with reference to the same experimental scenarios and combinations of resolutions.

In the case of the interaction analysis, poor resolutions strongly compromise interaction time values not revealing the difference between experiments with different biological conditions. The extracted mean interaction time distributions for the simulated control and the simulated treatment at extreme combinations of resolutions, i.e., (0.33 μm/pixel; 3 frames/min), (0.33 μm/pixel; 0.25 frames/min), (1.32 μm/pixel; 3 frames/min), and (1.32 μm/pixel; 0.25 frames/min), are shown in Fig. 5a. At lower resolutions, the crucial role of the tracking algorithm consisted in equalizing the distributions until they become almost uniforms, and above all reducing separability between them. At (1.32 μm/pixel; 0.25 frames/min) the discrimination ability is purely random (Fig. 5b).

In Supplementary Figure S4, we show the p-values maps obtained with K-S test when the simulated control was compared with different comparative scenarios, which mimic different biological conditions. By increasing the modulus of the drift and the effectiveness time to |***μ***| = 3.4 *μm*/*min* and *T*_*eff*_ = 11.6 min, respectively, (see Supplementary Fig. S4a), p-values higher than 0.05 interested a larger portion of the map than increasing *T*_*eff*_ only (see Supplementary Fig. S4b), with a similar trend obtained by varying |***μ***| only (see Supplementary Fig. S4c). The reason of this effect is that immune cells with a higher value of |***μ***| remain within the interaction radius for less time, leading the values of the mean interaction time to get closer to the values in the simulated control. On the contrary, by increasing even more *T*_*eff*_ in the abovementioned simulated treatment, p-values higher than 0.05 were resolved at very lower resolutions (see Supplementary Fig. S4d). Therefore, based on the evidence of the compared scenarios and on the chosen descriptor, the combined effect of spatial and temporal resolutions may result in failures to detect existing differences between experimental conditions. As a final remark, it is crucial to discuss about the choice of spatio-temporal resolution, when the experimental environment does not permit the optimal setting. Spatial resolution is the core point in the cell detection step. For the first two spatial resolutions, we obtained reasonable results in terms of detected cell trajectories and swapping error (see Supplementary Text S1 and Fig. S1). Practitioners may take into account the possibility to lose a large amount of cell trajectories if they want to acquire images at lower resolutions. For a scenario showing interaction, a higher number of missed cell trajectories leads to an increasing error in mean interaction time computation (Fig. S2b) and to a reduction in number of interacting cells (Fig. 3b). Temporal resolution also plays a fundamental role in the computation of mean interaction time as well as of kinematic descriptors. A low frame rate does not allow to capture a detailed cellular dynamic. Moreover, combined effects of spatio-temporal resolution may influence the evaluation of differences among diverse experimental conditions in terms of extracted descriptors. From the earlier discussion about Fig. S4, we underlined how these combined effects impacted differently on the grounds of the couple of experiments under examination.

In conclusion, practitioners have to carefully consider the experiments under observation also according to their own experience. It would be unpractical to suggest a unique setting for all the experiments, regardless if it is a problem of cell-cell interaction under cell subtyping (e.g., cells exhibit different receptors leading to weak different kinematics) or cell-cell interaction under different chemical stimuli that lead to highly different cell behaviour (e.g., immune and cancer cells in presence of not of chemotherapeutic agent). One possibility could be to run preliminary experiments using two different settings and see whether numerical results of the extract descriptors remain stable and statistical significance is verified. If so, consider the opportunity to use, for the following experiments, the less expensive configuration so to reduce computational time and memory required. Otherwise, the more compelling setting is required.

### Analysis of organ-on-chip experiments

**Figure 6:**
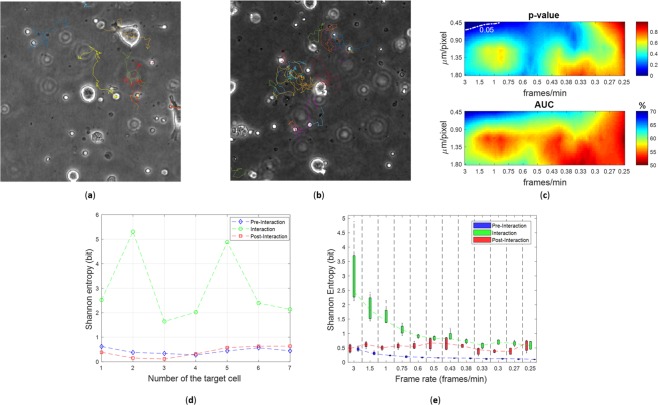
Organ-on-chip experiments. (**a**) Automatic tracking applied on a ROI for the control case experiment. (**b**) Automatic tracking applied on a ROI for the comparative case experiment. (**c**) Maps of p-values for the K-S test and the area under the receiver characteristic (AUC) curve at varying resolutions for the descriptor of interaction time. The white dotted line in the p-value maps indicates the contour line at p = 0.05. (**d**) Shannon entropy of angular speed behaviour for the comparative case in the three different motion phases (pre-interaction, interaction and post-interaction phase) averaged over all tracks of the immune cells in each of ROIs (Regions Of Interest). (**e**) Shannon Entropy boxplots for the three phases of motion: pre- interaction (blu), interaction (green), post-interaction (red) at fixed maximum spatial resolution and at decreasing temporal resolutions.

As experimental validation on videos from real cell co-cultures, we investigated how temporal and spatial resolutions affect the reliability of results extracted from two organ-on-chip experiments by exploiting tumor-immune on chip models in 3D settings. We performed the analysis on time-lapse videos acquired at spatio-temporal resolution of (0.45 μm/pixel; 3 frames/min) (see paragraph *Organ-on-chip experiment*). For the control and comparative cases, respectively, we extracted six and seven Regions Of Interest (ROIs), each of which focused on the interaction between immune cells and a single target cell. Each target cell was placed at the center of the video crop (see Supplementary Videos S6–S11 for the control case and Supplementary Videos S12–S18 for the comparative case), so that each ROI corresponds to one target cell. For the real case, we conducted the same analysis steps as for the simulated videos. Firstly, by considering the extracted ROIs as single videos, they were subsampled at different combinations of resolutions over the ranges [0.45–1.80] μm/pixel and [3–0.25] frames/min. Next, the automatic tracking software was applied. In Fig. 6a,b the extracted trajectories at maximum spatial and temporal resolutions for two ROIs (control case in Fig. 6a and comparative case in Fig. 6b) are shown. At the maximum resolutions, the statistical difference between the two scenarios was evident only in terms of interaction time (Fig. 6c), not in terms of kinematic descriptors. The result makes sense in light of the treatment adopted for the comparative case, which affected proliferation and survival of the target cells as well as the immune-cancer cells interaction, but it seems to have no effects or impact on immune cells kinematics (see paragraph *Organ-on-chip experiment*). The maps shown in Fig. 6c demonstrate that, by decreasing both spatial and temporal resolutions, the interaction time parameter loses its discriminative capacity. The information about the interaction, in fact, was lost when resolutions became lower, similarly to the case of the simulated scenarios (Fig. 3). In fact, by computing the Shannon entropy of the angular speed on the tracks of each of the seven ROIs (seven target cells) of the comparative case, during the interaction phase of the motion, the Shannon entropy values are clearly higher than the values of the pre- and post-interaction phases (Fig. 6c). Fixed the maximum spatial resolution, at decreasing temporal resolutions, the gap among the Shannon entropy distributions in the interaction phase and the distributions in the pre- and post-interaction phases drastically decreases (Fig. 6d).

## Methods

### Stochastic particle model

We use a two-dimensional stochastic particle model which describes immune cells migrating towards a target cell (cancer cell). During their random directed motion, immune cells are attracted by the cancer cell within an interaction radius for apriori imposed time. The model takes into account two distinct fundamental parts: (1) single-cell migration, (2) cell-cell interaction.

#### Single-cell migration

We model single immune cell dynamics using a random walk with drift^3^ and a dissipative damping term^27^. Random walks, however, are uncorrelated and independent from the presence of other immune cells. Therefore, we added to the model also cell-cell interaction components, as follows.

#### Cell-cell interaction

We distinguish two kinds of interactions: (1) immune-immune interaction as a short-scale repulsion, (2) cancer-immune interaction which includes an attraction component in addition to the repulsive component. The interactions are expressed as forces readapted to our case from^27^, where the authors intended to simulate a collective migration behaviour of a single cell population.

### Mathematical description of the model

With the aim of describing single immune cell dynamics, we approximated the motion of each cell by the motion of the corresponding geometric center. Denoting with ***r***_*i*_ the two-dimensional position vector of the *i*^*th*^ center, with Δ*t*_***i***_ the interval between two consequent time steps, the updating displacement, Δ***r***_*i*_ = ***r***_*i*_(*t*_*i*_) − ***r***_*i*_(*t*_*i*_ − 1), is given by

1 $$\Delta {{\boldsymbol{r}}}_{{\boldsymbol{i}}}={\mathscr{N}}(0,{{\boldsymbol{\sigma }}}_{i}){\rm{\Delta }}{{t}_{i}}^{1/2}+({{\boldsymbol{\mu }}}_{i}+{{\boldsymbol{v}}}_{i}^{d}){\rm{\Delta }}{t}_{i},$$

where the first term represents the random component of the displacement, modelled as a Gaussian random walk^3^, with volatility ***σ***_*i*_, whilst the second term, represents a deterministic component, with ***μ***_*i*_ a systematic drift and ${{\boldsymbol{v}}}_{i}^{d}$ a deterministic term varying with time. The deterministic drift, ***μ***_*i*_, outlines a general flow, deviated because of the presence of the cancer cell attracting the immune cell under consideration for an imposed time, so that, after interaction, the immune cell goes away from the interaction radius tacking the flow back^6^. In order to simulate a global flow, the drift was implemented with constant modulus contrary to its direction that, for the sake of simplicity, varies at each time from cell to cell according to the angle formed by the positions of the cell and the cancer cell. Since we are primarily interested in the study of how the interaction phenomenon is affected by changing spatial-temporal resolutions, in this way we ensure all cells will interact with the cancer cell. The deterministic speed, ${{\boldsymbol{v}}}_{i}^{d}$ is updated at each time step as

2 $${\partial }_{t}{{\boldsymbol{v}}}_{i}^{d}=-\,\alpha {{\boldsymbol{v}}}_{i}^{d}+\sum _{i\ne j}{{\boldsymbol{F}}}_{ij}+{\boldsymbol{f}},$$

where ∂_*t*_ denotes the temporal partial derivative. The first term expresses a dissipative damping with coefficient *α*^27^, whilst the other terms describe the interaction forces with cells belonging to the same population, $\sum _{i\ne j}{{\boldsymbol{F}}}_{ij}$, and with the target cell, ***f***, respectively. In detail, every addendum ***F***_*ij*_ = −∇*U*(*r*_*ij*_) denotes the force exerted to the *i*^*th*^ immune cell by the *j*^*th*^ immune cell at the distance *r*_*ij*_ = |***r***_***i***_ − ***r***_***j***_|. The term *U*(*r*_*ij*_), inspired by^27^, represents a short-scale Gaussian repulsive potential defined as,

3 $$U({r}_{ij})=\{\begin{array}{cc}{U}_{0}\exp (-{({r}_{ij}/{a}_{0})}^{2}), & \,{r}_{ij} < {R}_{im}\\ 0, & otherwise,\end{array}$$

where *U*_0_, *a*_0_, and *R*_*im*_ specify the intensity of the repulsive potential, the maximum immune cell-cell overlapping distance, and the interaction radius between the *i*^*th*^ and the *j*^*th*^ immune cells, respectively.

In addition, computed the distance ${r}_{i}^{c}$ of the *i*^*th*^ immune cell from the target cell, the interaction force between them is defined as^27^

4 $${\boldsymbol{f}}=-\,\nabla U({r}_{i}^{c}),$$

with

5 $$U({r}_{i}^{c})=\{\begin{array}{cc}{U}_{0}\exp (\,-\,{({r}_{i}^{c}/{a}_{1})}^{2})+{U}_{1}{({r}_{i}^{c}-{a}_{1})}^{2}H({r}_{i}^{c}-{a}_{1}), & \,{r}_{i}^{c} < {R}_{int}\\ 0, & otherwise.\end{array}$$

The interacting potential, $U({r}_{i}^{c})$, takes into account a repulsive potential (first term) similarly to equation (3) and a long-range attractive potential (second term). Given the Heaviside function, *H*(*x*), the attractive potential, whose intensity is *U*_1_, plays a role only when the *i*^*th*^ immune cell exceeds the interaction radius, *R*_*int*_, but it does not overlap the cancer cell $({r}_{i}^{c} > {a}_{1})$, as shown in Fig. 1A. The attractive potential acts for an imposed time that we call the effectiveness time, *T*_*eff*_. The motion of the tumor cell was modelled as a pure random walk.

### Numerical simulations

We integrate equation (2) explicitly using the Adams–Bashforth method^28^. The model parameters were chosen using realistic values mimicking the experiments in^15^ and then converted at a spatial resolution of 0.33 *μm*/*pixel* and a temporal resolution of 3 *frames*/*min*, which is six times faster than the frame rate used for most of our studies^5,6,15^. A region of interest of dimensions 198 *μm* × 198 *μm* was considered to mimic the last 80 *min* of the experiment in which *N* = 16 immune cells with radius *r*_*im*_ = 4 *μm*, and a cancer cell with radius *r*_*c*_ = 10 *μm* were present. Every *i*^*th*^ cell started its migration with a drift constant in modulus, ***μ***_*i*_, directed towards the target cell. The two components of ***σ***_*i*_ and *α* were set to 6.86 *μm*/*min*^2^ and 1.5 *min*^−1^,respectively. We imposed *R*_*im*_ = 2*r*_*im*_ and *R*_*int*_ = 2(*r*_*im*_ + *r*_*c*_); *U*_0_ and *U*_1_ were set as in^27^, whilst *a*_0_ = *R*_*im*_, *a*_1_ = *R*_*int*_/2. The cancer-immune attraction was exerted for a priori imposed time, named the effectiveness time, *T*_*eff*_. By varying *T*_*eff*_, thereby implicitly varying the interaction time between immune cells and the cancer cell, we were able to simulate different experimental conditions, e.g., not treated versus treated cells. For the control case, parameters were set in agreement with the experiments in^5,15^, with |***μ***| = 3 *μm*/min and *T*_*eff*_ = 8.3 *min* (see Supplementary Video S1). The alternative scenarios included different combinations of effectiveness time and of the modulus of the drift, i.e.,|***μ***| = 3.4 *μm*/*min* and *T*_*eff*_ = 8.3 *min* (see Supplementary Video S2), |***μ***| = 3.0 *μm*/*min* and *T*_*eff*_ = 11.6 *min* (see Supplementary Video S3), |***μ***| = 3.4 *μm*/*min* and *T*_*eff*_ = 11.6 *min*, (see Supplementary Video S4) |***μ***| = 3.0 *μm*/*min* and *T*_*eff*_ = 13.3 *min* (see Supplementary Video S5).

An atlas of 100 videos was generated in MATLAB R2017b (MathWorks, Natick, MA) as illustrated in Fig. 1B. Examples of generated video sequences are available in Supplementary Materials (see Supplementary Videos S1–S5). We thus obtained a population of *N*_*TOT*_ = 1600 cells, statistically useful to perform the analysis of the kinematic descriptors at different combinations of temporal and spatial resolutions. All videos were subsampled at different combinations of resolutions over the ranges [0.33–1.32] μm/pixel and [3–0.25] frames/min.

### Organ-on-chip experiment

Cancer cells (the HER2+ breast cancer BT474 cell line) and immune cells (PBMCs, peripheral blood mononuclear cells, from healthy donors) were embedded into 3D biomimetic hydrogels (made of collagen type I at 2.5 mg/mL, the major component of the extra-cellular matrix (ECM)), inside micro-chambers (1000 μm wide, 200 μm high) of microfluidic devises fabricated by soft lithography using PDMS (poly-dymethylsiloxane), a silicone rubber which is biocompatible, gas-permeable and transparent^5^. The targeted final densities of BT474 and PBMC cells in gels were respectively 1 × 10^6^ cells/mL and 10 × 10^6^ cells/mL (ration 1:10). The co-culture medium was EBM-2 (Lonza, CC-3156) supplemented with a cocktail of growth factors (Lonza, CC-4176).

PBMCs, peripheral blood mononuclear cells, were isolated from blood of healthy donors, obtained from the national blood bank “Etablissement francais du sang” (EFS), a public organization that is in charge of collecting, preparing, distributing blood derivatives mainly for transfusion purposes, strictly following French guidelines and regulations on the matter.

Time-lapse videos were acquired with a Leica DMi8 inverted microscope equipped with a Retiga R6 camera and illumination by Lumencor SOLA SE 365. The automated imaging system was controlled by the software Metamorph (Universal Imaging). Transmission images were taken every 20 sec for 3 hrs, at a spatial resolution of 0.454 μm/pixel.

### Statistical analysis

In the present work, we compare couple of diverse experimental conditions. To quantify how the difference between the two distributions reneges at decreasing resolutions we used two approaches. The first one is a statistical approach based on the Kolmogorov-Smirnov (K-S) test^29^, a nonparametric test that quantifies the difference between two empirical distribution functions with no assumption on normality of data. Under the null hypothesis that the samples are drawn from the same distributions, the obtained p-values quantify the strength of evidence against the null hypothesis. We considered here that p-values lower than 0.05 indicate a statistically significant difference between the two samples.

The second approach is based on the concept of predictive performance of the descriptors with respect to a binary classification problem in which the two classes are represented by the two experimental scenarios. Given the two classes, class1 (negative) and class2 (positive), e.g. corresponding to a control case and a comparative case, the Receiver Operating Characteristics (ROC) curve is a two-dimensional graph representing pairs of (sensitivity, 1- specificity) values for decreasing cut-points. The cut-points are used on the given descriptor as threshold values to determine the assigned class. Sensitivity expresses the rate of instances properly assigned to the positive class for a given cut-point, whilst 1-specificity indicates the rate of instances belonging to the negative class but assigned to the positive class, normalized over the actual number of positive and negative instances, respectively^30,31^. The Area Under the ROC Curve (AUC)^30^ provides information about the discriminatory ability of the descriptor: it can take values ranging from 0.5 to 1, indicating random guess and perfect discriminatory ability, respectively. The greater the AUC is (i.e., the more the curve approaches the top of the chart), the greater the discriminating power of the descriptor is.

## Supplementary information

Supplementary Info

Video S1

Video S2

Video S3

Video S4

Video S5

Video S6

Video S7

Video S8

Video S9

Video S10

Video S11

Video S12

Video S13

Video S14

Video S15

Video S16

Video S17

Video S18
