# Supplementary material for: The influence of spatial and temporal resolutions on the analysis of cell-cell interaction: a systematic study for time-lapse microscopy applications
Source: Sci Rep. 2019 May 1;9:6789. doi: 10.1038/s41598-019-42475-5 (PMC6494897; doi:10.1038/s41598-019-42475-5)
Supplement: Supplementary file 1 — Supplementary Info [file 41598_2019_42475_MOESM1_ESM.docx]

SUPPLEMTARY INFORMATION

The influence of spatial and temporal resolutions on the analysis of cell-cell interaction:

a systematic study for time-lapse microscopy applications

M. C. Comes^1^, P. Casti^1^, A. Mencattini^1^, D. Di Giuseppe^1^, F. Mermet-Meillon^2^, A. De Ninno^3,4^,

M. C. Parrini^2^, L. Businaro^4^, C. Di Natale^1^, E. Martinelli^1^

^1^ Department of Electronic Engineering, University of Rome Tor Vergata, Rome, Italy

^2^ Institute Curie, Centre de Recherche, Paris Sciences et Lettres Research University, 75005 Paris, France;

^3^ Department of Civil Engineering and Computer Science, University of Rome Tor Vergata, 00133 Rome, Italy

^4^ Institute for Photonics and Nanotechnology, Italian National Research Council, 00156 Rome, Italy;

* Corresponding author

E-mail: martinelli@ing.uniroma2.it

Associate Professor,

Dept. Electronic Engineering

University of Rome Tor Vergata

Via del politecnico 1, 00133 Roma, Italia

tel: +39 0672597259

**The tracking algorithm and performance evaluation at varying resolutions**

The tracking algorithm used in our previous studies, *Cell Hunter*, [1,2] was applied to the artificial videos at different resolutions for detecting cell candidates at each frame and for linking the detected cells along the frames (see Fig 1C). The detection stage is based on the segmentation of circular-shaped objects using the Circular Hough Transform (CHT) [3] with radius and tolerance tuned around the theoretical radius of immune cells at each spatial resolution. With the intent to study cell-cell interaction, we avoided to detect immune cells inside the target cells by masking it at each frame before immune cells are detected. Localized cells at the i^th^ frame are linked to cells located at the (i+1)^th^ frame by solving an Optimal Subpattern Assignment Problem (OAP) using the Munkres algorithm [4]. The algorithm yields the globally best possible pairing among located objects based on a given assignment cost equal to the inverse of the distance between pairs of cells at the i^th^ and (i+1)^th^ frames. In order to reduce the computational time and facilitate the linking task, only cells within a distance less than $R_{max}=5 r_{im}$ , with $r_{im}$ the radius of the immune cells, between subsequent frames are considered during the assignment problem.

The algorithm accounts for cell appearing/disappearing and cell occlusion, therefore the number of incoming and outcoming cells is not forced to be the same. Three different situations may occur:

- Incoming and outcoming cells are the same number, and a one-to-one assignment is expected.
- Incoming cells are more than the outcoming cells. In this case, some cells irremediably will not be linked to any outcoming cell. Such cells remain in a *standby situation* of duration *t_s_*. This solution accounts for temporal occlusion/disappearing of the cells. Those cells can be then linked to other cells in the next *t_s_* frames or otherwise stop definitely their track.
- Incoming cells are less than outcoming cells. In this case, some outcoming cells will not be the continuation of any previous track. Hence, with the aim to account for cell appearing (i.e., cell entering the videos) such cells are marked as starting track cells.

Detected trajectories with a duration less than half of the total number of frames are rejected, as it happens in practice, to guarantee an adequate number of data point within each trajectory for the analysis.

We used the number of detected trajectories and the swapping error as indices of performance [5] at varying resolutions. The tracking algorithm could fail in particle tracking in two ways: by missing the trajectories, as shown in S1A Fig, and by associating tracts corresponding to different detected trajectories to a single trajectory (see S1C Fig). We show in S1B Fig that the percentage of detected trajectories over the $1600$ total theoretical trajectories, decreased when spatial and temporal resolutions worsen. The same behaviour was observed for the number of cell candidates detected by the CHT because the recognition of circular objects becomes more difficult as the spatial resolution decreases whilst, for the case of the temporal resolution, the candidates decrease as a reduced number of available frames with the same video. A different trend characterized the number of swaps per track, which increases with decreasing spatial resolutions and decreases with decreasing temporal resolutions (see S1D Fig). The observed trend is expected since, at larger frame rates, cells are more detached from frame to frame and swapping is less probable. The maximum number of swaps is localized in correspondence of the resolutions combination (1.32 μm/pixel; 3 frames/min), when the assignment problem becomes more difficult.

x

| 1. | Parlato, S. *et al.*, 3D Microfluidic model for evaluating immunotherapy efficacy by tracking dendritic cell behaviour toward tumor cells. *Scientific Reports* **7** (1), 1093 (2017). |
| --- | --- |
| 2. | Biselli, E. *et al.*, Organs on chip approach: a tool to evaluate cancer-immune cells interactions. *Scientific Reports* **7** (1), 12737 (2017). |
| 3. | Davies, E. R., *Machine vision: theory, algorithms, practicalities* (Elsevier, 2004). |
| 4. | Munkres, J., Algorithms for the assignment and transportation problems. *Journal of the society for industrial and applied mathematics* **5** (1), 32-38 (1957). |
| 5. | Huth, J. *et al.*, Significantly improved precision of cell migration analysis in time-lapse video microscopy throygh use of a fully automated tracking system. *BCM cell biology* **11** (1), 24 (2010). |

x

**Figure Captions**

**Supplementary Figure S1.** **Performance of the cell tracking algorithm.** (a) Example of missed (not detected) trajectory, shown with green asterisks, at 0.33 μm/pixel and 0.25 frames/min. Detected (coloured) and ground truth (black dots) trajectories are also indicated. (b) Percentage of detected trajectories at decreasing spatial and temporal resolutions. (c) Example of switch in the trajectory. (d) Average number of swaps per trajectory at decreasing spatial and temporal resolutions. Mean values in (b) and (c) are reported with markers while error bars indicate the standard deviation values over the 100 simulations.

(TIF)

**Supplementary Figure S2.** Mean (left column) and standard deviation (right column) maps of the relative error at varying spatial and temporal resolution for different feature descriptors. For the computation of the relative error, the i^th^ feature extracted from each trajectory detected in the j^th^ video, 𝑓_𝑖𝑗_, is compared with the corresponding values extracted using the ground-truth trajectories 𝑔_𝑖𝑗_, as ${Err}_{ij}=\left| \left\langle f_{ij} \right\rangle-\left\langle g_{ij} \right\rangle\right|/\left| \left\langle g_{ij} \right\rangle\right|$, where 〈∙〉 denotes the ensemble average on all the trajectories detected in the jt^h^ video. (a) MSD. (b) Interaction time. (c) Migration speed.

(TIF)

**Supplementary Figure S3.** Comparison of diverse experimental conditions for other kinematic descriptors in addition to those in Fig. 4. (a) Mean interaction time. (b) Mean angular speed. (c) Shannon entropy of the angular speed. (d) Migration Speed. In the legends S.C. denotes the simulated control (|𝝁| = 3.0 𝜇𝑚/𝑚𝑖𝑛, 𝑇_𝑒𝑓𝑓_ = 8.3 min), S.T. denotes the simulated treatment (|𝝁|=3𝜇𝑚/𝑚𝑖𝑛, 𝑇_𝑒𝑓𝑓_=13.3 min). Left panels represent the feature values (mean and standard deviation values) at the maximum spatial resolution (0.33 𝜇𝑚/𝑝𝑖𝑥𝑒𝑙) and decreasing temporal resolution. Central panels represent the feature values at the maximum temporal resolution (3 frames/min) and decreasing spatial resolutions. Every region delimited by vertical dotted lines stands for a unique temporal (left panel) or spatial (central panel) resolution. Right panels show the maps of p-values for the K-S test and the area under the receiver characteristic (AUC) curve at varying resolutions. The white dotted line in the p-value maps indicates the contour line at p = 0.05.

(TIF)

**Supplementary Figure S4.** Maps of p-values for the K-S test obtained for different couples of experimental scenarios simulating various realistic biological conditions. The control scenario: $\left| \boldsymbol{\mu} \right|=3.0 \mu m/min$, $T_{eff}=8.3 min,$ is compared with different scenarios: (a) $\left| \boldsymbol{\mu} \right|=3.4 \mu m/min$, $T_{eff}=11.6 min,(b$) $\left| \boldsymbol{\mu} \right|=3.0 \mu m/min$, $T_{eff}=11.6 min,(c$) $\left| \boldsymbol{\mu} \right|= 3.4 \mu m/min$, $T_{eff}=8.3 min$, and (d) $\left| \boldsymbol{\mu} \right|=3.0 \mu m/min$, $T_{eff}=13.3 min$. The white dotted line indicates the contour line at p = 0.05.

(TIF)


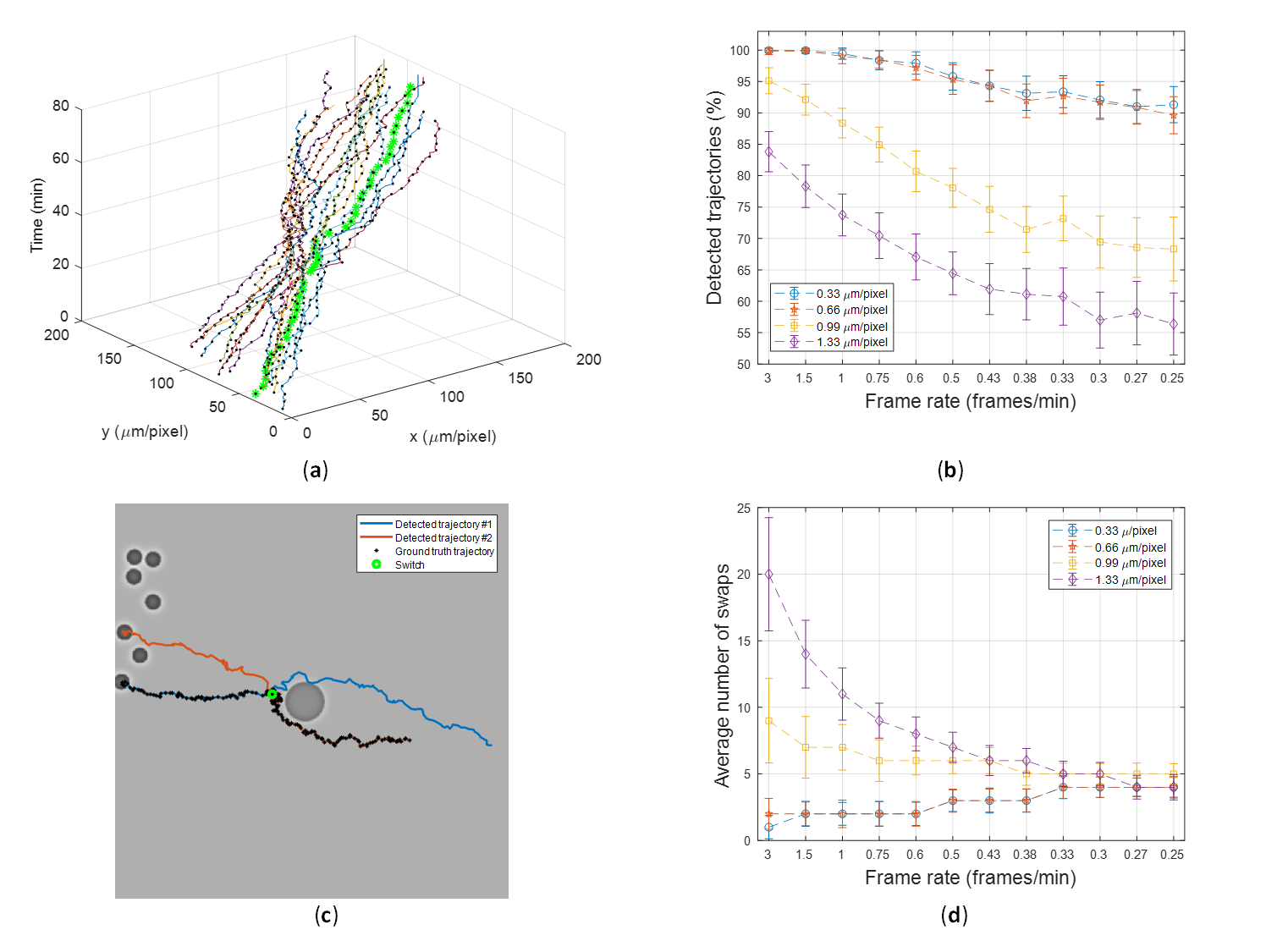


**Figure S1**

**
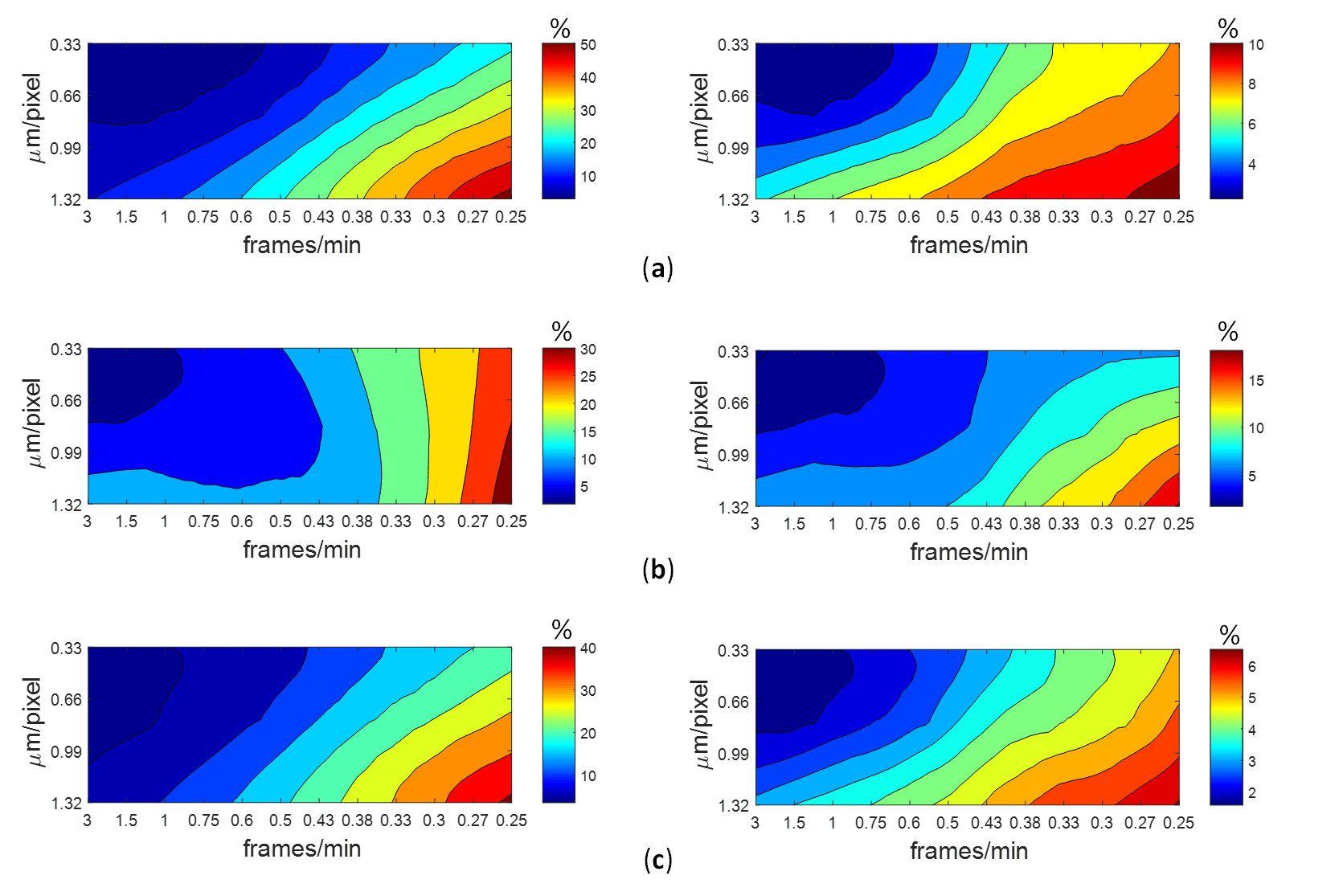
**

**Figure S2**

**
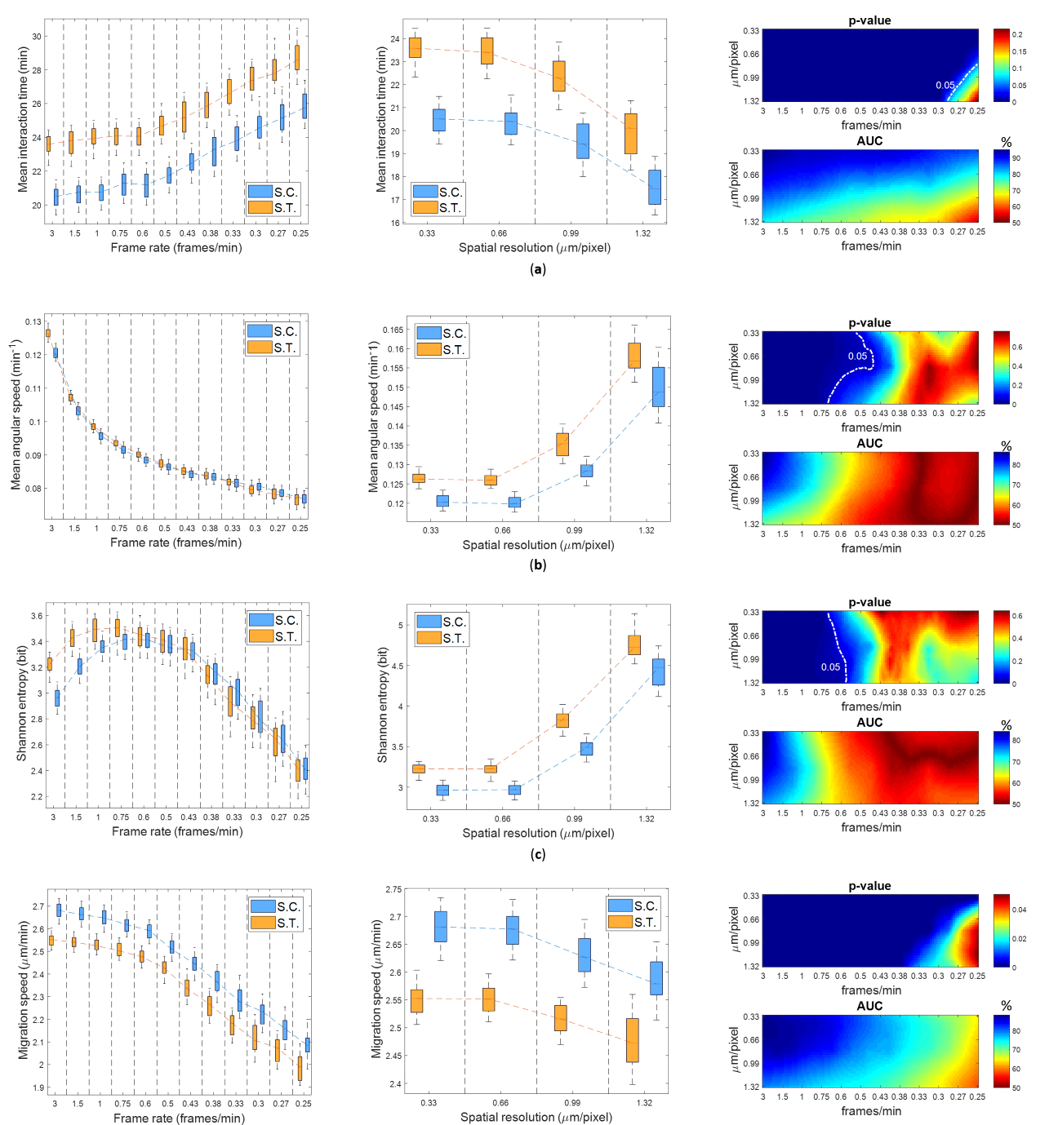
**

**Figure S3**

**
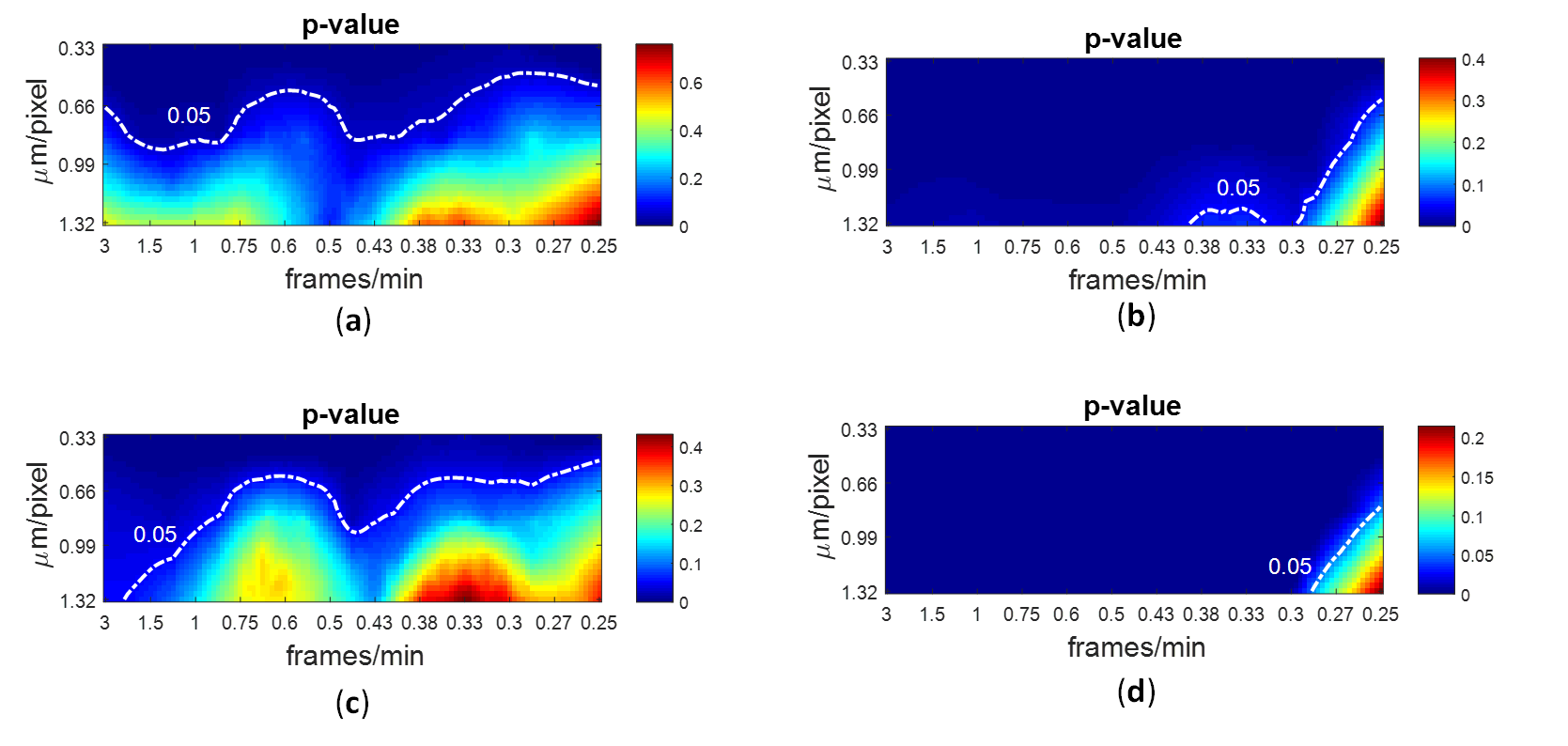
**

**Figure S4**

**All the videos are available on the following link:**

<https://www.dropbox.com/sh/7wzrzdh6p914zox/AAAtYJs5SZo6KwqWpuUJxjWRa?dl=0>

**Supplementary Video S1.** Movie of one out of 100 mimicking the motion of the immune particles towards the target cell by imposing motion constraints of $\left| \boldsymbol{\mu} \right|=3.0{\mu m}/{min}$ for the drift modulus and $T_{eff}=8.3 min$ for the effectiveness time.

(AVI)

**Supplementary Video S2.** Movie of one out of 100 mimicking the motion of the immune particles towards the target cell by imposing motion constraints of $\left| \boldsymbol{\mu} \right|=3.4 \mu m/min$ for the drift modulus and $T_{eff}=8.3 min$ for the effectiveness time.

(AVI)

**Supplementary Video S3.** Movie of one out of 100 mimicking the motion of the immune particles towards the target cell by imposing motion constraints of $\left| \boldsymbol{\mu} \right|=3.0 \mu m/min$ for the drift modulus and $T_{eff}=11.6 min$ for the effectiveness time.

(AVI)

**Supplementary Video S4.** Movie of one out of 100 mimicking the motion of the immune particles towards the target cell by imposing motion constraints of $\left| \boldsymbol{\mu} \right|=3.4 \mu m/min$ for the drift modulus and $T_{eff}=11.6 min$ for the effectiveness time.

(AVI)

**Supplementary Video S5.** Movie of one out of 100 mimicking the motion of the immune particles towards the target cell by imposing motion constraints of $\left| \boldsymbol{\mu} \right|=3.0 \mu m/min$ for the drift modulus and $T_{eff}=13.3 min$ for the effectiveness time.

(AVI)

**Supplementary Video S6-S11.** Extracted Regions of Interest (ROIs) for the control case of the OOC real experiment.

(AVI)

**Supplementary Video S12-S18.** Extracted Regions of Interest (ROIs) for the comparative case of the OOC real experiment.

(AVI)
